# Association of cardiovascular-kidney-metabolic syndrome with all-cause and cardiovascular mortality: A prospective cohort study

**DOI:** 10.1016/j.ajpc.2025.100985

**Published:** 2025-03-29

**Authors:** Jiangtao Li, Xiang Wei

**Affiliations:** a Division of Cardiovascular Surgery, Tongji Hospital, Tongji Medical College, Huazhong University of Science and Technology, Wuhan, PR China; b Key Laboratory of Organ Transplantation, Ministry of Education, Wuhan, PR China; c NHC Key Laboratory of Organ Transplantation, Ministry of Health, Wuhan, PR China

**Keywords:** Cardiovascular-kidney-metabolic syndrome, Mortality, Prospective cohort study

## Abstract

**Background:**

Given evidence on the cardiovascular disease (CVD) risk conferred by comorbidity risk factors, the American Heart Association (AHA) recently introduced a novel staging construct, named cardiovascular-kidney-metabolic (CKM) syndrome. This study examined the association of CKM syndrome stages with all-cause and cardiovascular mortality among US adults.

**Methods:**

Data were from the National Health and Nutrition Examination Survey (NHANES) 1999–2018 at baseline linked to the 2019 National Death Index records. For each participant, the CKM syndrome was classified into five stages: stage 0 (no CKM risk factors), 1 (excess or dysfunctional adiposity), 2 (metabolic risk factors and chronic kidney disease), 3 (subclinical CVD), or 4 (clinical CVD). The main outcomes were all-cause and cardiovascular mortality.

**Results:**

Among 34,809 participants (mean age: 46.7 years; male: 49.2 %), the prevalence of CKM stages 0 to 4 was 13.2 %, 20.8 %, 53.1 %, 5.0 %, and 7.8 %, respectively. During a median follow-up of 8.3 years, compared to participants with CKM stage 0, those with higher stages had increased risks of all-cause mortality (stage 2: HR 1.43, 95 % 1.13–1.80; stage 3, HR 2.75, 95 % CI 2.12–3.57; stage 4, HR 3.02, 95 % CI 2.35–3.89). The corresponding hazard ratios (95 % confidence interval) of cardiovascular mortality risks were 2.96 (1.39–6.30), 7.60 (3.50–16.5), and 10.5 (5.01–22.2). The population-attributable fractions for advanced (stages 3 or 4) vs. CKM syndrome stages (stages 0, 1, or 2) were 25.3 % for all-cause mortality and 45.3 % for cardiovascular mortality.

**Conclusion:**

Higher CKM syndrome stages were associated with increased risks of all-cause and cardiovascular mortality. These findings emphasize that primordial and primary prevention efforts on promoting CKM health should be strengthened to reduce mortality risk.

## Graphical abstract

Cardiovascular-kidney-metabolic syndrome stages and the risk of mortality

**Figure:**
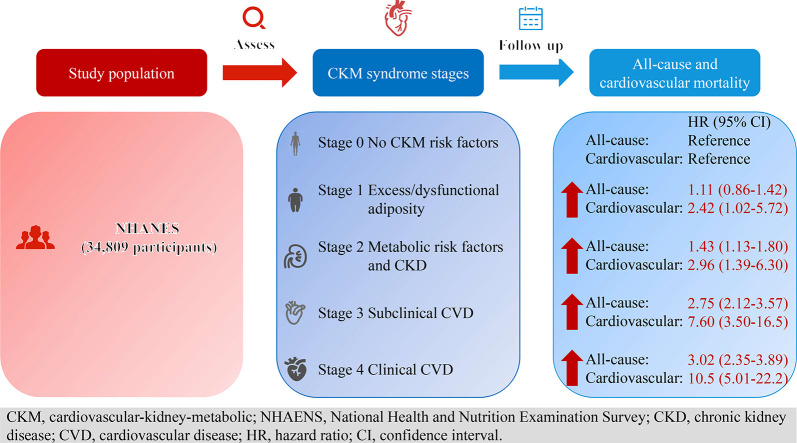

## 1 Introduction

Cardiovascular disease (CVD), chronic kidney disease (CKD), and metabolic disease represent three major health challenges that contribute to substantial morbidity and mortality [1]. According to a report from the American Heart Association (AHA), CVD accounted for roughly 19.1 million global deaths in 2020 [2]. It was reported that 1.2 million people died from CKD in 2017 and there was a 41.5 % (95 % uncertainty interval 35.2 to 46.5) increase in the global all-age mortality rate due to CKD between 1990 and 2017 [3]. Additionally, 529 million people were living with diabetes worldwide, and the global age-standardized total diabetes prevalence was 6.1 % in 2021 [4].

Epidemiological findings from observational studies and clinical trials have provided substantial evidence of the significant overlap between cardiovascular, kidney, and metabolic diseases [[5], [6], [7], [8]]. Acknowledging this intricate relationship, the AHA introduced a novel framework named cardiovascular-kidney-metabolic (CKM) syndrome,[9] for enhancing multidisciplinary approaches to prevention, risk stratification, and management of these disorders. Classified by risk factors and established disease, the CKM syndrome was divided into five stages, including stage 0 (no CKM risk factors), stage 1 (excess or dysfunctional adiposity), stage 2 (metabolic risk factors and CKD), stage 3 (subclinical CVD), and stage 4 (clinical CVD). To the best of our knowledge, no studies have assessed the performance of different CKM syndrome stages with all-cause and cardiovascular mortality; these data may inform healthcare design, research, and policy efforts. From a public health standpoint, it is of great importance to validate the utility of CKM syndrome stages to promote awareness and adherence to the current recommendations, thus potentially reducing the burden of CKM syndrome and related mortality.

Accordingly, based on nationally representative data and using the novel classification framework, we examined the association of CKM syndrome stages with all-cause and cardiovascular mortality among US adults. Furthermore, we calculated the population-attributable fractions (PAF) of CKM syndrome stages in relation to mortality risk.

## 2 Methods

### 2.1 Study design and population

This study used data from the National Health and Nutrition Examination Survey (NHANES). The NHANES is an ongoing, national, cross-sectional survey with a complex, stratified, and multistage probability sampling design of the noninstitutionalized US civilian population. The NHANES was approved by the National Center for Health Statistics Research Ethics Review Board, and written informed consent was obtained from all participants. This study was exempt from review because it involved secondary data analysis of deidentified data, which poses minimal risk to participants’ privacy. This study adhered to the Strengthening the Reporting of Observational Studies in Epidemiology (STROBE) reporting guideline [10].

**Fig. 1:**
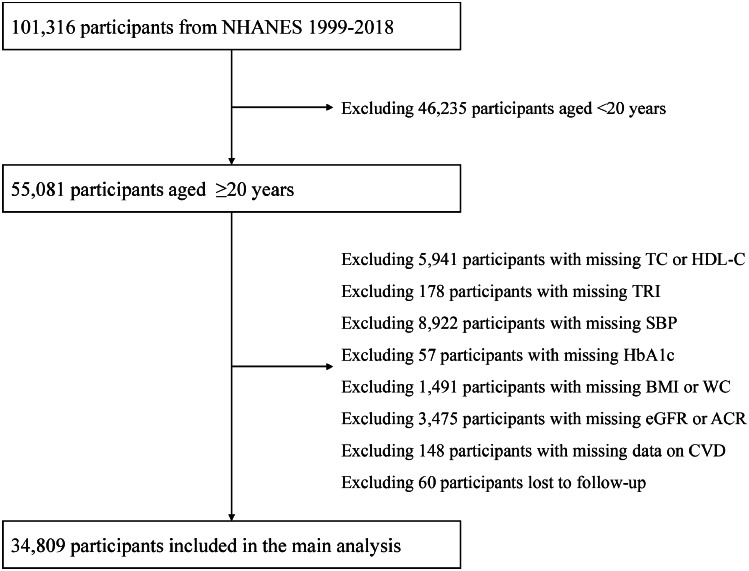
Selection process of the study population.

NHANES, National Health and Nutrition Examination Survey; TC, total cholesterol; HDL-C, high-density lipoprotein cholesterol; TRI, triglyceride; SBP, systolic blood pressure; HbA1c, glycosylated hemoglobin A1c; BMI, body mass index; WC, waist circumference; eGFR, estimated glomerular filtration rate; ACR, urinary albumin to creatinine ratio; CVD, cardiovascular disease.

This study used data from 10 NHANES cycles (from 1999–2000 to 2017–2018). Fig. 1 shows the selection process of the study population. After excluding participants with the age of < 20 (n = 46,235), those with missing data on determining CKM syndrome stage (*n* = 20,212), and those lost to follow-up (n = 60), a total of 34,809 adults aged ≥20 years were included in this study.

### 2.2 Definitions of CKM syndrome stages

CKM syndrome stages were defined based on the criteria from the 2023 American Heart Association (AHA) Presidential Advisory on CKM Health [9]. For each participant, the CKM syndrome was classified into five stages (stages 0 to 4). Stage 0 included individuals with normal body mass index (BMI, <23 kg/m^2^ for individuals with Asian ethnicity and <25 kg/m^2^ for other racial and ethnic groups), normal waist circumference (<80 and <90 cm for women and men with Asian race, respectively, and <88 and <102 cm for women and men in all other races, respectively), normoglycemia, normotension, a normal lipid profile, and without metabolic risk factors (diabetes, hypertension, hypertriglyceridemia, and no evidence of CKD or subclinical or clinical CVD). Stage 1 included individuals with an elevated BMI (≥23 kg/m^2^ for individuals of Asian race and >25 kg/m^2^ for all other races), an elevated waist circumference (≥80 and ≥90 cm for women and men with Asian race, respectively, and ≥88 and ≥102 cm for women and men in other races, respectively), or prediabetes (glycated hemoglobin of 5.7 % to <6.5 % or fasting blood glucose of 100 mg/dL to <126 mg/dL) without the presence of other metabolic risk factors or CKD. Stage 2 included individuals with metabolic risk factors (elevated fasting serum triglyceride levels ≥135 mg/dL, hypertension, diabetes, or metabolic syndrome) or moderate- to high-risk CKD per Kidney Disease Improving Global Outcomes (KDIGO) criteria [9]. Stage 3 included individuals with very high-risk CKD per KDIGO criteria or a high (≥20 %) predicted 10-year CVD risk [11]. Stage 4 included individuals with clinical CVD, including coronary heart disease, angina, heart attack, heart failure, or stroke. The CKD stages were classified based on the estimated glomerular filtration rate (eGFR, calculated using the race-free CKD Epidemiology Collaboration 2021 creatinine equation[12]) and urinary albumin to creatinine ratio. The 10-year CVD risk was estimated with the AHA Predicting Risk of CVD EVENTs (PREVENT) equations [11]. The PREVENT equations were firstly developed for variables with the following ranges: age 30–79 years, total cholesterol (TCH) 130–320 mg/dL, high-density lipoprotein cholesterol (HDL-C) 20–100 mg/dL, systolic blood pressure (SBP) 90–200 mmHg, BMI 18.5-<40 kg/m^2^, and eGFR 15–150 mL/min/1.73m². Therefore, the 10-year CVD risk was not estimated for adults out of the values. However, to minimize underestimation of CKM stage 3, participants with values exceeding the threshold were not excluded from the 10-year CVD risk. To approximate PREVENT risk strata, values for these variables above or below these bounds were set to the upper or lower bounds of allowable values respectively (for example, age 90 years was set as 79 years). Advanced CKM stages were defined as stages 3 or 4 because these identify individuals at high risk of CVD or with established CVD, nonadvanced CKM stages were defined as stages 0, 1, or 2.

### 2.3 Definition of mortality

Baseline data from NHANES 2005–2018 were merged with mortality data from the National Death Index death certificate records until December 31, 2019, matched using a probabilistic matching algorithm to identify mortality status. The outcomes included all-cause mortality and cardiovascular mortality (codes I00–I09, I11, I13, I20–I51, and I60–I69) using the International Classification of Disease Tenth Revision.

### 2.4 Assessment of covariates

The covariates included age, gender, race and ethnicity, education, marital status, family income to poverty ratio, labor force status, smoking status, drinking status, history of arthritis, chronic lung disease, and cancer, white blood cell (WBC), hemoglobin, platelets, blood urea nitrogen (BUN), and uric acid (UA). Race and ethnicity were divided into Mexican American, non-Hispanic Black, non-Hispanic White, other Hispanic, and other races. Education was classified into three levels: less than high school, high school, and college or above. Marital status was divided into two categories: married or living with a partner and other marital status (including separated, divorced, widowed, or unmarried). The family income to poverty ratio was classified into three levels: < 1.3, 1.3–<3.5, and ≥ 3.5. Labor force status was divided into working and not working. Smoking status was categorized as never, former, and current smokers. Similarly, drinking status was categorized as never, former, and current drinkers.

### 2.5 Statistical analyses

Data were adjusted for the complex sampling survey design of NHANES, with strata, primary sampling units, and probability weights incorporated into statistical models using survey analysis procedures. For normally distributed continuous variables, mean values and their standard deviations (SD) were reported. Categorical variables were described in terms of numbers and percentages. Differences in baseline characteristics across five CKM stages were assessed using survey-weighted linear regression for continuous variables and survey-weighted Pearson χ2 tests for categorical variables. Missing rates for covariates were summarized in Supplemental Table 1. Missing data of covariates were imputed using multiple imputations with chained equations [13]. We performed 5 imputations and generated 5 imputed datasets. Effect estimates were computed separately for each of the 5 datasets, and then combined according to Rubin's rules [13]. The multiple imputations were conducted using the R package "mice".

Kaplan–Meier survival curves were generated to calculate the survival probability of mortality using five CKM stages (stages 0 to 4), compared using the log-rank test. To analyze the association of CKM stages with the risk of all-cause and cardiovascular mortality, Cox proportional hazard regression was used to calculate the hazard ratio (HR) and its 95 % confidence interval (CI). Two models were fitted for the Cox regression using participants with CKM stage 0 as the reference. Model 1 was unadjusted. Model 2 was adjusted for age, gender, race and ethnicity, education, marital status, family income to poverty ratio, labor force status, smoking status, drinking status, history of arthritis, chronic lung disease, and cancer, WBC, hemoglobin, platelets, BUN, and UA. The adjusted PAFs of advanced (stages 3 to 4) vs. nonadvanced CKM stages (stages 0 to 2) were estimated for the proportion of all-cause and cardiovascular mortality associated with advanced CKM stages that could be avoided if all of those were at nonadvanced stages [14]. Furthermore, the adjusted PAFs of CKM stage 3 vs. nonadvanced stages (stages 0 to 2) were also estimated.

Several sensitivity analyses were conducted: (i) to minimize the reverse causality, the main analyses were repeated after excluding participants who died in the first two years of follow-up; (ii) we repeated the main analyses after excluding participants with continuous values outside of the ranges set by the AHA PREVENT equations; (iii) because the cancer was a major cause of mortality, the main analyses were repeated after excluding participant with a history of cancer; (iv) we repeated the main analyses after further adjusting for the uses of antihypertensive, antidiabetic, and lipid-lowering drugs; (v) considering the competing risk between non-cardiovascular mortality and cardiovascular mortality, we repeated the main analyses using the competing risk model [15]. Finally, stratified analyses were performed to examine the associations of CKM stages with all-cause and cardiovascular mortality by age, gender, race/ethnicity, education, marital status, family income to poverty ratio, and labor force status.

All statistical analyses were carried out by R software (Version 4.3.2). P-values were two-sided, and a P value < 0.05 was considered statistically significant.

## 3 Results

### 3.1 Baseline characteristics of the study population

This study analyzed data from 34,809 participants, 13.2 % (*n* = 3688) of participants were categorized in CKM stage 0, 20.8 % (*n* = 6575) in stage 1, 53.1 % (*n* = 18,233) in stage 2, 5.0 % (*n* = 2803) in stage 3, and 7.8 % (*n* = 3510) in stage 4 (Table 1). Among them, a weighted mean (SD) age was 46.7 (16.6) years, and a weighted 49.2 % were male and 50.8 % female. Compared to those with CKM stage 0, participants with higher stages (1 to 4) were more likely to be older, male, married or living with a partner, former smokers, never drinkers, have a history of arthritis, chronic lung disease, and cancer, and have a low education level and low family income to poverty ratio (all *P* < 0.001). Participants with higher CKM stages had higher levels of WBC, BUN, and UA as compared with participants with stage 0. In addition, we described the baseline characteristics of participants using data not being imputed (Supplemental Table 1). These results were similar to those presented in Table 1.

**Table 1:** Baseline characteristics of participants according to CKM syndrome stages using imputed data.

Variable	CKM syndrome					P value
	Stage 0	Stage 1	Stage 2	Stage 3	Stage 4	
Number ( %)	3688 (13.2)	6575 (20.8)	18,233 (53.1)	2803 (5.0)	3510 (7.8)	
Age, mean (SD), years	35.5 (12.6)	39.8 (13.6)	47.0 (14.3)	74.5 (8.1)	63.9 (13.3)	<0.001
Age group, *n* ( %)						<0.001
20 to <45 years	2913 (76.5)	4419 (65.4)	7558 (43.2)	29 (1.1)	263 (9.3)	
45 to <65 years	644 (20.6)	1770 (29.1)	7915 (44.5)	254 (8.7)	1168 (37.4)	
≥65 years	131 (2.9)	386 (5.5)	2760 (12.4)	2520 (90.2)	2079 (53.3)	
Gender, *n* ( %)						<0.001
Male	1487 (38.6)	2640 (42.2)	9302 (53.1)	1636 (53.0)	2053 (56.2)	
Female	2201 (61.4)	3935 (57.8)	8931 (46.9)	1167 (47.0)	1457 (43.8)	
Race and ethnicity, *n* ( %)						<0.001
Mexican American	465 (5.8)	1174 (9.4)	3633 (9.6)	358 (4.8)	432 (4.7)	
Non-Hispanic Black	301 (5.2)	627 (6.6)	1805 (5.8)	194 (3.8)	232 (3.5)	
Non-Hispanic White	1837 (73.0)	2565 (64.8)	7424 (67.7)	1502 (75.3)	1915 (74.9)	
Other Hispanic	627 (8.5)	1543 (12.8)	3433 (9.5)	566 (10.9)	697 (10.2)	
Other Race	458 (7.6)	666 (6.4)	1938 (7.4)	183 (5.3)	234 (6.7)	
Education, *n* ( %)						<0.001
Below high school	603 (11.4)	1345 (13.0)	4781 (16.9)	1008 (26.2)	1207 (24.3)	
High school	760 (19.6)	1376 (20.8)	4260 (24.3)	707 (28.1)	880 (27.7)	
College or above	2325 (69.0)	3854 (66.3)	9192 (58.8)	1088 (45.7)	1423 (48.0)	
Marital status, *n* ( %)						<0.001
Married/living with a partner	2013 (57.9)	3966 (64.3)	11,748 (67.6)	1580 (58.5)	2052 (63.5)	
Other marital status	1675 (42.1)	2609 (35.7)	6485 (32.4)	1223 (41.5)	1458 (36.5)	
Family income to poverty ratio, *n* ( %)						<0.001
Low (< 1.3)	1060 (19.8)	1950 (19.9)	5674 (20.6)	889 (23.2)	1299 (26.9)	
Middle (1.3–3.5)	1291 (32.7)	2417 (35.2)	6634 (34.4)	1314 (49.3)	1435 (41.3)	
High (>3.5)	1337 (47.4)	2208 (44.8)	5925 (45.0)	600 (27.4)	776 (31.8)	
Labor force status, *n* ( %)						<0.001
Working	2679 (76.4)	4779 (77.0)	11,387 (68.7)	433 (16.4)	778 (29.5)	
Not working	1009 (23.6)	1796 (23.0)	6846 (31.3)	2370 (83.6)	2732 (70.5)	
Smoking status, *n* ( %)						<0.001
Never smokers	2290 (60.6)	4084 (59.4)	10,038 (53.3)	1270 (45.4)	1371 (38.6)	
Former smokers	487 (15.0)	1195 (21.0)	4313 (25.4)	1129 (41.3)	1385 (38.9)	
Current smokers	911 (24.4)	1296 (19.7)	3882 (21.3)	404 (13.3)	754 (22.5)	
Drinking status, *n* ( %)						<0.001
Never drinkers	806 (17.3)	1627 (19.5)	4727 (20.5)	947 (33.4)	986 (25.6)	
Former drinkers	187 (4.2)	440 (6.6)	1979 (9.6)	625 (19.3)	882 (22.4)	
Current drinkers	2695 (78.5)	4508 (73.9)	11,527 (69.9)	1231 (47.3)	1642 (52.0)	
Arthritis, n ( %)	281 (8.9)	886 (14.8)	4612 (25.3)	1397 (52.1)	1952 (55.0)	<0.001
Chronic lung disease, n ( %)	126 (3.9)	264 (4.5)	1189 (6.9)	275 (10.9)	667 (20.1)	<0.001
Cancer, n ( %)	145 (5.0)	286 (5.3)	1263 (8.0)	660 (29.5)	686 (21.0)	<0.001
WBC, mean (SD), 10^9/L	6.7 (1.9)	6.9 (2.1)	7.5 (2.7)	7.4 (2.7)	7.6 (3.4)	<0.001
Hemoglobin, mean (SD), g/dL	14.1 (1.4)	14.1 (1.4)	14.5 (1.4)	13.9 (1.6)	14.1 (1.5)	<0.001
Platelets, mean (SD), 10^9/L	245.2 (59.7)	250.7 (60.2)	254.5 (64.8)	231.3 (65.1)	233.6 (70.8)	<0.001
BUN, mean (SD), mg/dl	12.1 (3.8)	12.4 (4.0)	13.2 (4.3)	19.2 (8.7)	16.7 (7.9)	<0.001
UA, mg/dL	4.6 (1.1)	5.0 (1.2)	5.6 (1.4)	5.9 (1.5)	5.9 (1.5)	<0.001

Categorical variables are presented as number (weighted percentage) and continuous variables as mean ± SD.

Differences in baseline characteristics across the five CKM syndrome stages were assessed using survey-weighted linear regression for continuous variables and survey-weighted Pearson χ2 tests for categorical variables.

CKM, cardiovascular-kidney-metabolic; SD, standard deviation; WBC, white blood cell; BUN, blood urea nitrogen; UA, uric acid.

### 3.2 Associations of CKM stages with all-cause and cardiovascular mortality

**Fig. 2:**
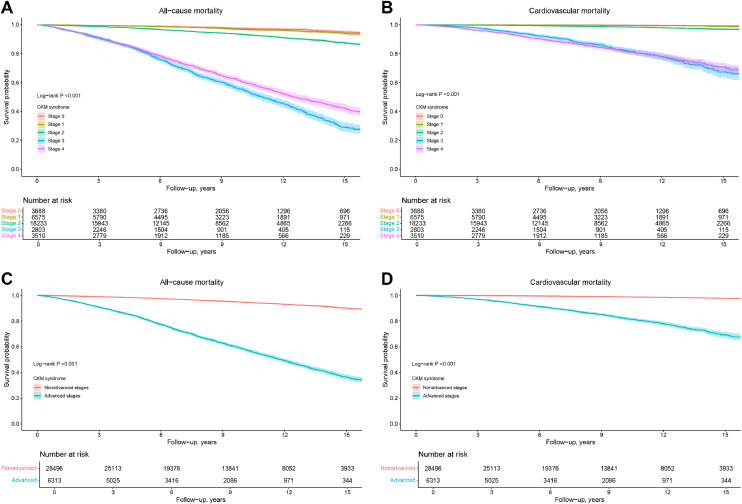
Kaplan–Meier curves for cumulative all-cause and cardiovascular mortality by cardiovascular-kidney-metabolic syndrome stages. (A-B) CKM stages 0 to 4; (C-D) Nonadvanced and advanced CKM stages.

During a median follow-up period of 8.3 years, out of the 34,809 participants with CKM syndrome, 4020 (11.5 %) died, with 1212 (3.5 %) deaths attributed to cardiovascular disease. The median time to death was 6.7 (IQR, 3.7–10.4) years for all-cause mortality and 6.7 (3.9–10.7) years for cardiovascular mortality. The Kaplan-Meier (K-M) survival analysis demonstrated significant differences in all-cause and cardiovascular mortality rates among the CKM syndrome stages mortality (*P* < 0.001 for all log-rank tests, Fig. 2). Compared with those with CKM stage 0, participants with higher stages had a significantly increased cumulative incidence rate of all-cause and cardiovascular mortality (Fig. 2A and 2B). Similarly, participants with nonadvanced CKM stages (stages 0–2) showed a better survival probability in terms of all-cause and cardiovascular mortality than those with advanced stages (stages 3–4) (Fig. 2C and 2D). In addition, compared with those with CKM stage 0, participants with higher stages had a significantly increased cumulative incidence rate of diabetes and CKD mortality (Supplemental Figure S1).

CKM, cardiovascular-kidney-metabolic.

Table 2 illustrates the Cox regression models examining the association between CKM syndrome stages and all-cause mortality. After adjusting for confounders, compared to participants with CKM stage 0, those with advanced CKM stages presented significantly increased risks of all-cause mortality (stage 3, HR 2.75, 95 % CI 2.12–3.57; stage 4, HR 3.02, 95 % CI 2.35–3.89). Meanwhile, significantly elevated risks of all-cause mortality were also found in participants with CKM stage 2 (HR 1.43, 95 % 1.13–1.80). The trend tests indicated that there were increasing trends of all-cause mortality risks with the increasing CKM stages (P for trend <0.001). In addition, we compared the adjusted PAF for each progressive CKM stage with the previous stage and found that the adjusted PAF of stage 3 vs. stage 2 was the highest (21.3 %). When dividing CKM stages into nonadvanced (stages 0 to 2) and advanced stages (stages 3 to 4), participants with advanced CKM stages presented significantly increased risks of all-cause mortality (HR 2.13, 95 % CI 1.91–2.38) compared to participants with nonadvanced stages. The adjusted PAF of advanced (stages 3 to 4) vs. nonadvanced CKM stages (stages 0 to 2) with all-cause mortality was 25.3 % (95 % CI 22.6 %−27.9 %). The adjusted PAF was 17.7 % (95 % CI 15.3 %−20.1 %) when comparing CKM stage 3 vs. nonadvanced CKM stages.

**Table 2:** Association of cardiovascular-kidney-metabolic syndrome stages with the risk of all-cause mortality.

CKM syndrome	Cases, n ( %)	HR1 (95 % CI)^a^	*P1*^a^	HR2 (95 % CI)^b^	*P2*^b^
Stage 0	107 (2.9)	1 (reference)		1 (reference)	
Stage 1	217 (3.3)	1.29 (1.02–1.65)	0.037	1.11 (0.86–1.42)	0.434
Stage 2	1279 (7.0)	2.80 (2.25–3.49)	<0.001	1.43 (1.13–1.80)	0.002
Stage 3	1168 (41.7)	26.5 (21.1–33.4)	<0.001	2.75 (2.12–3.57)	<0.001
Stage 4	1249 (35.6)	18.9 (14.9–24.0)	<0.001	3.02 (2.35–3.89)	<0.001
*P* for trend		<0.001		<0.001	
PAF1, %^c^				8.1 (−5.9–22.1)	0.258
PAF2, %^d^				11.0 (0.78–21.3)	0.035
PAF3, %^e^				21.3 (18.5–24.1)	<0.001
PAF4, %^f^				7.1 (4.0–10.2)	<0.001
Nonadvanced stages	1603 (5.6)	1 (reference)		1 (reference)	
Advanced stages	2417 (38.2)	10.17 (9.29–11.15)	<0.001	2.13 (1.91–2.38)	<0.001
PAF5, %^g^				25.3 (22.6–27.9)	<0.001
PAF6, %^h^				17.7 (15.3–20.1)	<0.001

a HR1 and P1 were unadjusted.

b HR2 and P2 were adjusted for age, gender, race and ethnicity, education, marital status, family income to poverty ratio, labor force status, smoking status, drinking status, history of arthritis, chronic obstructive pulmonary disease, and cancer, white blood cell, hemoglobin, platelets, blood urea nitrogen, and uric acid.

c PAF1 indicated the adjusted PAF of CKM stage 1 vs. stage 0.

d PAF2 indicated the adjusted PAF of CKM stage 2 vs. stage 1.

e PAF3 indicated the adjusted PAF of CKM stage 3 vs. stage 2.

f PAF4 indicated the adjusted PAF of CKM stage 4 vs. stage 3.

g PAF5 indicated the adjusted PAF of advanced (stages 3 to 4) vs. nonadvanced CKM stages (stages 0 to 2).

h PAF6 indicated the adjusted PAF of CKM stage 3 vs. nonadvanced CKM stages (stages 0 to 2). CKM, cardiovascular-kidney-metabolic; HR, hazard ratio; CI, confidence interval; PAF, population-attributable fraction.

Table 3 shows the Cox regression models assessing the relationship between CKM syndrome stages and cardiovascular mortality. After adjusting for confounders, compared to participants with CKM stage 0, those with advanced CKM stages presented significantly elevated risks of cardiovascular mortality (stage 3, HR 7.60, 95 % CI 3.50–16.5; stage 4, HR 10.5, 95 % CI 5.01–22.2). Meanwhile, significantly increased risks of cardiovascular mortality were also found in participants with CKM stage 2 (HR 2.96, 95 % 1.39–6.30). The trend tests indicated that there were increasing trends of cardiovascular mortality risks with the increasing CKM stages (P for trend <0.001). We also compared the PAF for each progressive CKM stage with the previous stage and found that the adjusted PAF of stage 3 vs. stage 2 was the highest (31.5 %). When dividing CKM stages into nonadvanced (stages 0 to 2) and advanced stages (stages 3 to 4), participants with advanced CKM stages presented significantly increased risks of cardiovascular mortality (HR 3.35, 95 % CI 2.70–4.16) compared to participants with nonadvanced stages. The adjusted PAF of advanced (stages 3 to 4) vs. nonadvanced CKM stages (stages 0 to 2) with cardiovascular mortality was 45.3 % (95 % CI 40.0 %−50.7 %). The adjusted PAF was 28.0 % (95 % CI 22.2 %−33.9 %) when comparing CKM stage 3 vs. nonadvanced CKM stages.

**Table 3:** Association of cardiovascular-kidney-metabolic syndrome stages with the risk of cardiovascular mortality.

CKM syndrome	Cases, n ( %)	HR1 (95 % CI)^a^	*P1*^a^	HR2 (95 % CI)^b^	*P2*^b^
Stage 0	13 (0.4)	1 (reference)		1 (reference)	
Stage 1	43 (0.7)	3.04 (1.30–7.10)	0.010	2.19 (0.93–5.18)	0.071
Stage 2	296 (1.6)	6.83 (3.25–14.4)	<0.001	2.96 (1.39–6.30)	0.005
Stage 3	363 (13.0)	99.9 (46.8–213.2)	<0.001	7.60 (3.50–16.5)	<0.001
Stage 4	497 (14.2)	82.9 (40.0–172.0)	<0.001	10.5 (5.01–22.2)	<0.001
*P* for trend		<0.001		<0.001	
PAF1, %^c^				8.1 (−5.9–22.1)	0.258
PAF2, %^d^				11.0 (0.78–21.3)	0.035
PAF3, %^e^				21.3 (18.5–24.1)	<0.001
PAF4, %^f^				7.1 (4.0–10.2)	<0.001
Nonadvanced stages	352 (1.2)	1 (reference)		1 (reference)	
Advanced stages	860 (13.6)	18.2 (15.4–21.6)	<0.001	3.35 (2.70–4.16)	<0.001
PAF5, %^g^				45.3 (40.0–50.7)	<0.001
PAF6, %^h^				28.0 (22.2–33.9)	<0.001

a HR1 and P1 were unadjusted.

b HR2 and P2 were adjusted for age, gender, race and ethnicity, education, marital status, family income to poverty ratio, labor force status, smoking status, drinking status, history of arthritis, chronic obstructive pulmonary disease, and cancer, white blood cell, hemoglobin, platelets, blood urea nitrogen, and uric acid.

c PAF1 indicated the adjusted PAF of CKM stage 1 vs. stage 0.

d PAF2 indicated the adjusted PAF of CKM stage 2 vs. stage 1.

e PAF3 indicated the adjusted PAF of CKM stage 3 vs. stage 2.

f PAF4 indicated the adjusted PAF of CKM stage 4 vs. stage 3.

g PAF5 indicated the adjusted PAF of advanced (stages 3 to 4) vs. nonadvanced CKM stages (stages 0 to 2).

h PAF6 indicated the adjusted PAF of CKM stage 3 vs. nonadvanced CKM stages (stages 0 to 2). CKM, cardiovascular-kidney-metabolic; HR, hazard ratio; CI, confidence interval; PAF, population-attributable fraction.

Supplemental Figure S2 shows the relationship between other covariates and all-cause mortality. Older age, non-Hispanic White, other marital status, not working, current smokers, former drinkers, the presence of chronic lung disease and cancer, and higher levels of WBC and UA are associated with increased risks of all-cause mortality. Female and higher levels of education, family income to poverty ratio, hemoglobin, and platelets are associated with lower risks of all-cause mortality. Similar results are observed for the association between covariates and cardiovascular mortality (Supplemental Figure S3).

### 3.3 Sensitivity analyses

After excluding participants who died in the first two years of follow-up, the relationship between CKM syndrome stages and mortality remained consistent (Supplemental Tables 3–4). Participants with higher CKM stages had increased risks of all-cause and cardiovascular mortality than participants with stage 0. The associations of CKM syndrome stages with all-cause and cardiovascular mortality were also consistent after excluding participants with continuous values outside of the ranges set by the AHA PREVENT equations (Supplemental Tables 5–6), further adjusting for the uses of antihypertensive, antidiabetic, and lipid-lowering drugs (Supplemental Tables 7–8), or excluding participants with a history of cancer (Supplemental Tables 9–10). Furthermore, the results were consistent with the main analyses after conducting the competing risk analyses between non-cardiovascular mortality and cardiovascular mortality (Supplemental Table 11). The stratified analyses were conducted to assess the robustness of the regression results on the relationship between CKM syndrome stages and all-cause and cardiovascular mortality. Similar results were observed across subgroups of age, sex, race/ethnicity, education, marital status, family income to poverty ratio, and labor force status (Supplemental Tables 12–25).

## 4 Discussion

In this prospective study of a nationally representative sample of US adults, we examined the associations of CKM syndrome stages with all-cause and cardiovascular mortality risks. Participants with CKM stages 2 to 4 had an increased risk of all-cause and cardiovascular mortality than participants with stage 0. Our adjusted PAF estimates suggested that 25.3 % of all-cause mortality and 45.3 % of cardiovascular mortality risk associated with advanced CKM stages could be avoided/eliminated if all of those were in nonadvanced CKM stages. Our findings lend support to the novel staging construct of CKM syndrome due to its utility in the prediction of future all-cause and cardiovascular mortality.

According to a report of Heart Disease and Stroke Statistics-2023 Update,[2] among US adults, the prevalence estimates indicate 9.9 % for CVD (comprising coronary heart disease, heart failure, and stroke), 15 % for CKD, and 13 % for diabetes, with approximately 95 % of diabetes attributed to type 2 diabetes. While individually considered, each of these three conditions is associated with relevant morbidity and mortality, but it is broadly recognized that they often coexist. Previous have reported that patients with heart failure have a four-fold higher prevalence of diabetes (20 %) than those without heart failure (4–6 %),[16] and diabetes is associated with a 2 to 4-fold higher risk of developing CVD [17]. In addition, a recent study reported a CKD prevalence of nearly 40 % among individuals with T2D and 50 % among individuals with HF [18]. Conversely, CVD is more frequently diagnosed among patients with CKD than in the general population, as its prevalence is inversely related to kidney function [19]. In recognition of this intricate relationship, the AHA has recently introduced the concept of a combined disease condition known as CKM syndrome, highlighting the close associations and interplay between these three health domains [20]. Our analysis of the NHANES dataset demonstrated the prevalence of individuals meeting each stage of the CKM syndrome diagnostic criteria among US adults. This study found that more than 85 % of adults met the criteria for CKM syndrome (stage 1 or higher), and 12.8 % met the criteria for advanced CKM stages (stages 3 or 4). These results highlight the high burden of risk factors for CVD, including among individuals who have not yet developed clinical CVD, and the low prevalence of individuals without CKM syndrome.

Based on a newly defined staging scheme recommended for CKM syndrome, we found that higher CKM stages were associated with an increased risk of all-cause and cardiovascular mortality. Understanding the mortality risk of different CKM stages is important for public health efforts behind the management of CKM syndrome, particularly given the increasing therapeutic management options for these conditions. These initiatives include targeted screening and prevention efforts among individuals with CKM stage 0 to prevent progression to higher stages or clinical CVD. Our adjusted PAF estimates suggested that 17.7 % of all-cause mortality and 28.0 % of cardiovascular mortality risk associated with CKM stage 3 could be avoided/eliminated if all of those were in nonadvanced CKM stages (stages 0 to 2). Several studies have found that hypertension, diabetes, metabolic syndrome, and CKD were associated with significantly higher risks of adverse outcomes [4,9,21,22]. For example, a large cohort study of 36,414 adults indicated that, compared with those without metabolic syndrome, individuals with metabolic syndrome had 1.2- and 1.4-times risk of all-cause and cardiovascular mortality, respectively [22]. Our study revealed that participants with CKM stage 3 had higher prevalence of these conditions (Supplemental Table 26), which may partly explain the significantly increased risks of all-cause and cardiovascular mortality. In addition, previous studies have reported that multiple cardiovascular risk factors are associated with the risk of CVD and mortality [[23], [24], [25], [26]]. A meta-analysis study of forty-two trials including 144,220 patients suggested that groups with a mean SBP of 120 to 124 mm Hg had a reduced risk of all-cause mortality (HR 0.59, 95 % CI 0.45–0.77) and major CVD (HR 0.58, 95 % CI 0.48–0.72) compared with groups with a mean SBP of 140 to 144 mm Hg [27]. In a large cohort study of 1120,295 US adults, Go et al. [26] reported that the risks of mortality and cardiovascular events increased as the eGFR decreased below 60 ml per minute per 1.73 m^2^ of body-surface area. In our study, participants with CKM stage 3 had higher levels of BMI, waist circumference, SBP, HbA1c, TCH, triglycerides, and eGFR, while lower levels of HDL-C than those with nonadvanced CKM stages (Supplemental Table 26). A prospective cohort study of 360,202 Chinese with type 2 diabetes indicated that CKD, CVD, suboptimal SBP, suboptimal HbA1c, and suboptimal weight were the leading attributable risk factors for all-cause and cardiovascular mortality [28]. In addition, the PAFs of these risk factors for cardiovascular mortality were significantly higher than those for all-cause mortality [28]. Similarly, in our study, participants with CKM stage 3 had higher prevalence of abovementioned risk factors, resulting in a higher PAF for cardiovascular mortality than for all-cause mortality. Among participant who might now have subclinical CVD (stage 3) in CKM syndrome, some may be unable to attain lower stages due to functional or other limitations, yet the improvements in CKM stages can still enhance their relative risk profile. For instance, better control of blood pressure and blood lipid levels, as well as quitting smoking, can reduce the predicted 10-year CVD risk and improve the CKM stage, resulting in a decreased mortality risk. That is some improvements are better than none.

In this study, the observed association of CKM stages with mortality was more pronounced among younger adults than older adults. The association between the exposure of risk factors and adverse outcomes is related to the exposure intensity and life stage. Several studies have found that young-onset hypertension,[29] diabetes[30], and metabolic syndrome,[31] are associated with increased risks of adverse clinical outcomes. A cohort study of 24,675 participants found that despite a lower absolute risk of heart failure, the population attributable risks of risk factors, such as hypertension, diabetes, obesity, and current smoking history, were higher in young compared with elderly adults [32]. Chen et al. [33] in a recent study of 471,269 participants from the UK Biobank suggested that associations of most risk factors and biomarkers with type 2 diabetes were markedly attenuated with increasing age at onset. In addition, a prospective cohort of 28,024 US showed that diabetes, hypertension, obesity, and smoking appeared to be the strongest risk factors for premature onset of coronary heart disease [34]. Furthermore, most risk factors and biomarkers showed attenuated associations at older ages [34]. Therefore, our findings highlight the importance of prevention strategies for CKM syndrome throughout the adult life course, with a great focus on the identification and management of risk factors among young people.

Overweight and obese individuals are usually complicated with hypertension, diabetes, hyperlipidemia, CKD, and CVD, which contribute to substantial mortality [35,36]. A meta-analysis of 239 prospective studies suggested that the associations of both overweight and obesity with higher all-cause mortality were broadly consistent in four continents [37]. However, our results showed that CKM stage 1, with more than 85 % being overweight or obese, was not significantly associated with all-cause mortality compared to stage 0. In this study, CKM stage 1 included individuals with an elevated BMI or an elevated waist circumference without the presence of other metabolic risk factors, CKD, or subclinical or clinical CVD. This finding may explain that the role of obesity in all-cause mortality was mediated by obesity-related chronic conditions. As for cardiovascular mortality, a large cohort study of 425,394 adults reported that overweight and obesity were associated with a progressively higher risk of cardiovascular mortality in younger adults, while they were not significantly associated with cardiovascular mortality in older adults [38]. Similarly, our findings showed that CKM stage 1 was only significantly associated with an increased cardiovascular mortality risk among young adults (Supplemental Table 13). Future research with larger sample sizes and longer follow-up are needed to determine the impact of CKM stage 1 on mortality.

Our study has important implications for clinical practice, public health, and future research. First, the results of this study contribute to the literature by providing robust evidence of the link between CKM syndrome stages and all-cause and cardiovascular mortality in US adults. These findings highlight the importance of early identification and intervention for individuals with CKM syndrome to mitigate the risk of adverse outcomes. Second, integrating the assessment of CKM stages into routine clinical practice is essential. Individuals with advanced CKM stages should be considered as the primary target to prevent all-cause and cardiovascular mortality. Furthermore, individuals with nonadvanced CKM stages also need to evaluate the risk factors so that at-risk individuals can be identified early and the corresponding prevention measures can be performed to delay the disease progression and reduce the mortality risk.

This study has several strengths. To the best of our knowledge, this is the first study to investigate the association between CKM syndrome stages and the risk of all-cause and cardiovascular mortality. Furthermore, this study included a large-scale, nationally representative cohort from different ethnicities, featuring rigorous study designs and large sample sizes. The diverse sensitivity analyses further substantiated the robustness of our results.

There are some limitations in this study. First, only clinical characteristics available in the NHANES dataset could be included in this analysis. The recommended data to define advanced CKM syndrome stages, including cardiac biomarkers, echocardiography, coronary angiography, cardiac computed tomography, atrial fibrillation, and peripheral artery disease, were unavailable, which may lead to underestimation of stages 3 and 4. Second, self-reporting of medical history could influence the prevalence estimates of CKM syndrome stages. Third, although we adjusted for multiple confounders, residual confounding might remain, such as genetic susceptibility and diet. Fourth, selection bias might occur in the main analyses because we excluded 20,272 participants due to missing data on determining CKM stages or lost to follow-up. The differences of baseline characteristics between included and excluded participants for the main analyses (Supplemental Tables 27–28) may affect the generalizability of the findings to the entire U.S. adult population. Finally, we only used measures of CKM syndrome stages in a single visit, and we did not consider the progression or improvement of CKM stages over time. This static assessment may influence the results, as changes in CKM stages—driven by lifestyle modifications, pharmacological interventions, or other factors during follow-up—can significantly impact the prognosis of participants. Consequently, this limitation may lead to either underestimation or overestimation of the true association between CKM stages and mortality outcomes.

## 5 Conclusions

Our study reveals a high prevalence of CKM syndrome (stages 1 to 4) among US adults, and higher CKM stages were associated with an increased risk of all-cause and cardiovascular mortality. These findings emphasize that primordial and primary prevention efforts on promoting CKM health should be strengthened to reduce mortality risk.

## Funding

None

## Data sharing statement

Data from the National Health and Nutrition Examination Survey (NHANES) are publicly available online.

## CRediT authorship contribution statement

**Jiangtao Li:** Writing – review & editing, Writing – original draft, Methodology, Investigation, Formal analysis, Data curation, Conceptualization. **Xiang Wei:** Writing – review & editing, Supervision, Resources, Project administration, Conceptualization.

## Declaration of competing interest

The authors declare that they have no known competing financial interests or personal relationships that could have appeared to influence the work reported in this paper.

## Appendix Supplementary materials

Image, application 1

Image, application 2
