# Supplementary material for: Association of cardiovascular-kidney-metabolic syndrome with all-cause and cardiovascular mortality: A prospective cohort study
Source: Am J Prev Cardiol. 2025 Mar 29;22:100985. doi: 10.1016/j.ajpc.2025.100985 (PMC12003006; doi:10.1016/j.ajpc.2025.100985)
Supplement: Supplementary file 1 [file mmc1.docx]

**Supplemental Figure S1** Kaplan–Meier curves for cumulative diabetes and CKD mortality by CKM syndrome stages. (A-B) CKM stages 0 to 4; (C-D) Nonadvanced and advanced CKM stages.


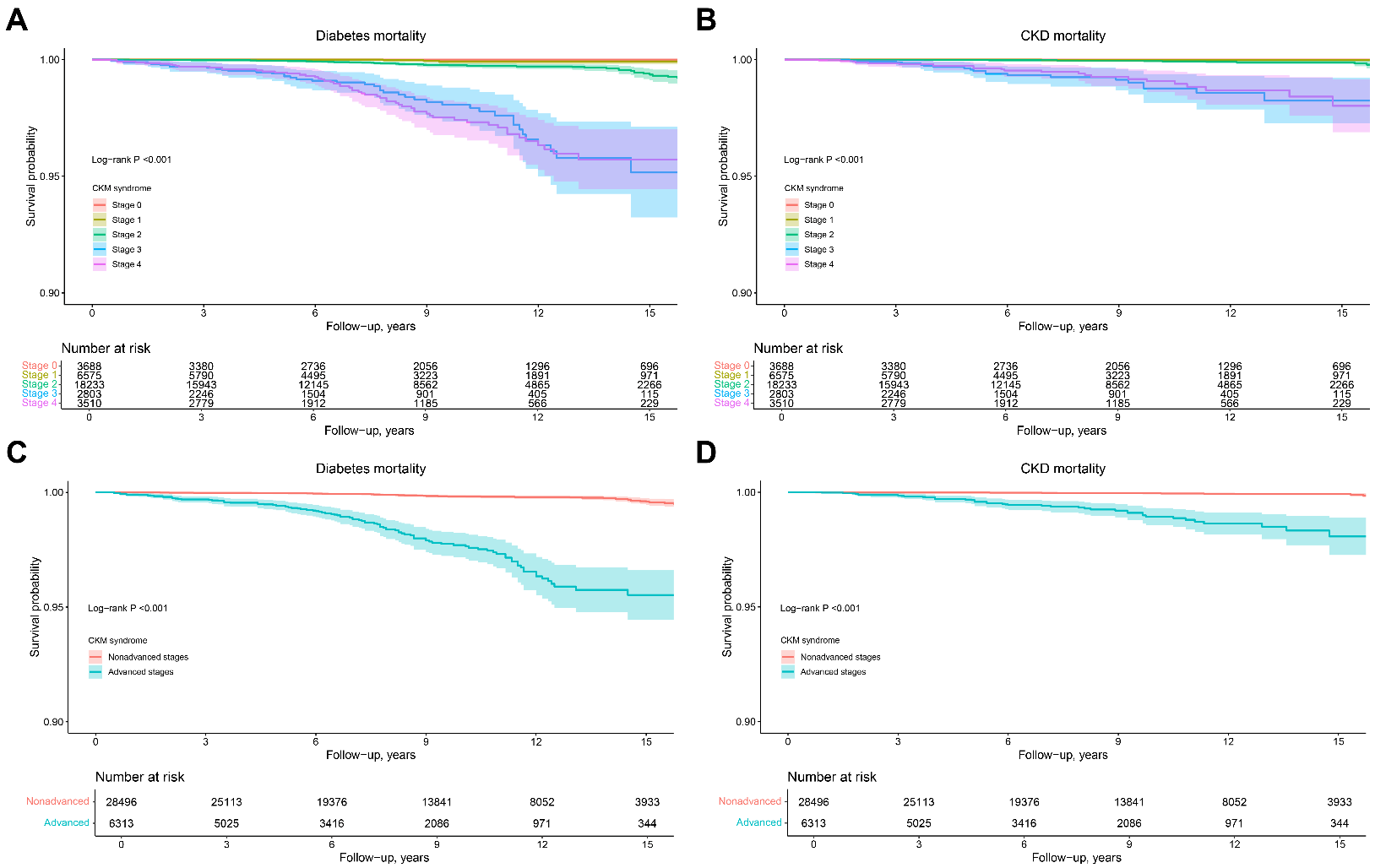


CKD, chronic kidney disease; CKM, cardiovascular-kidney-metabolic.

**Supplemental Figure S2** Association of covariates in this study with the risk of all-cause mortality among adults with CKM syndrome.


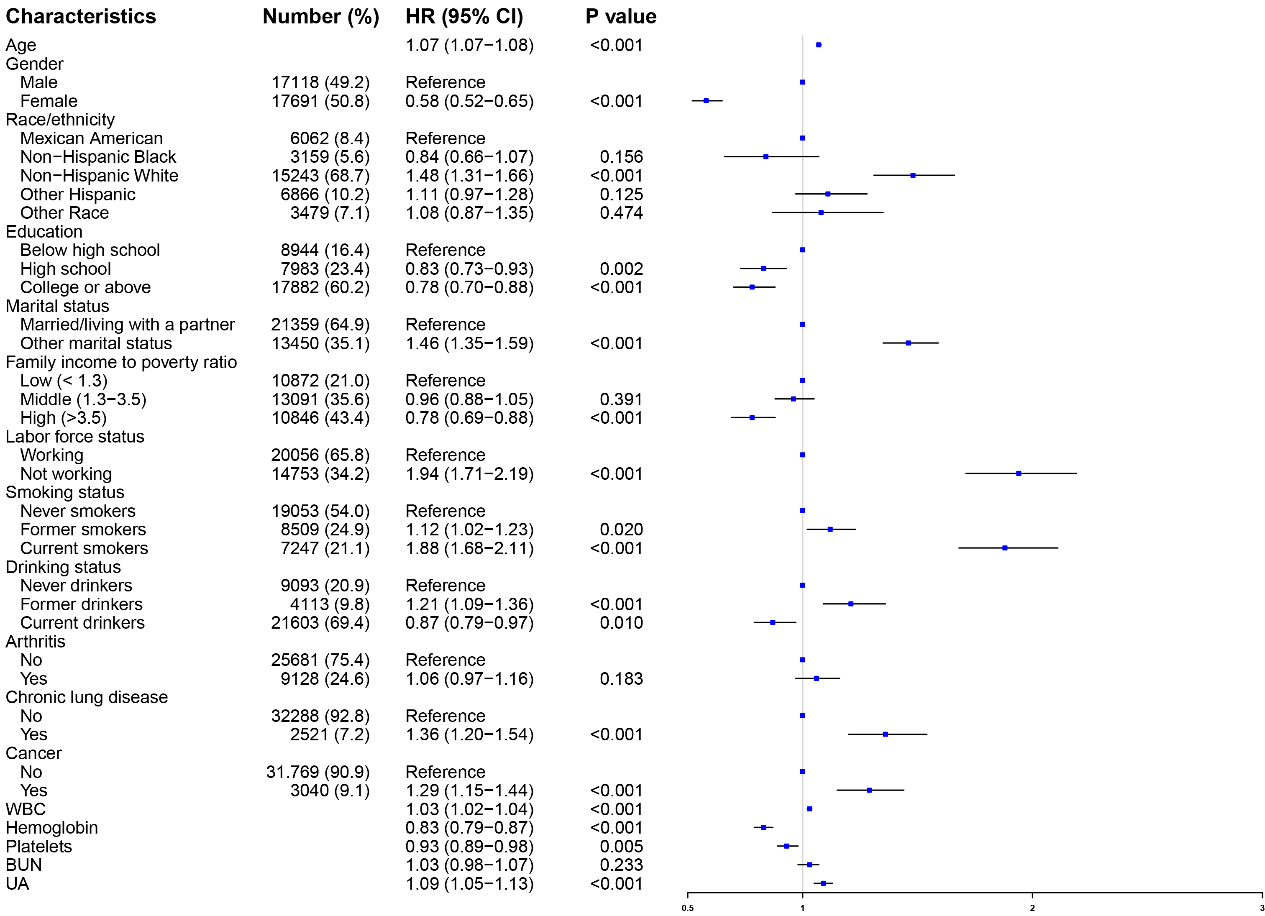


CKM, cardiovascular-kidney-metabolic; HR, hazard ratio; CI, confidence interval; WBC, white blood cell; BUN, blood urea nitrogen; UA, uric acid.

**Supplemental Figure S3** Association of covariates in this study with the risk of cardiovascular mortality among adults with CKM syndrome.


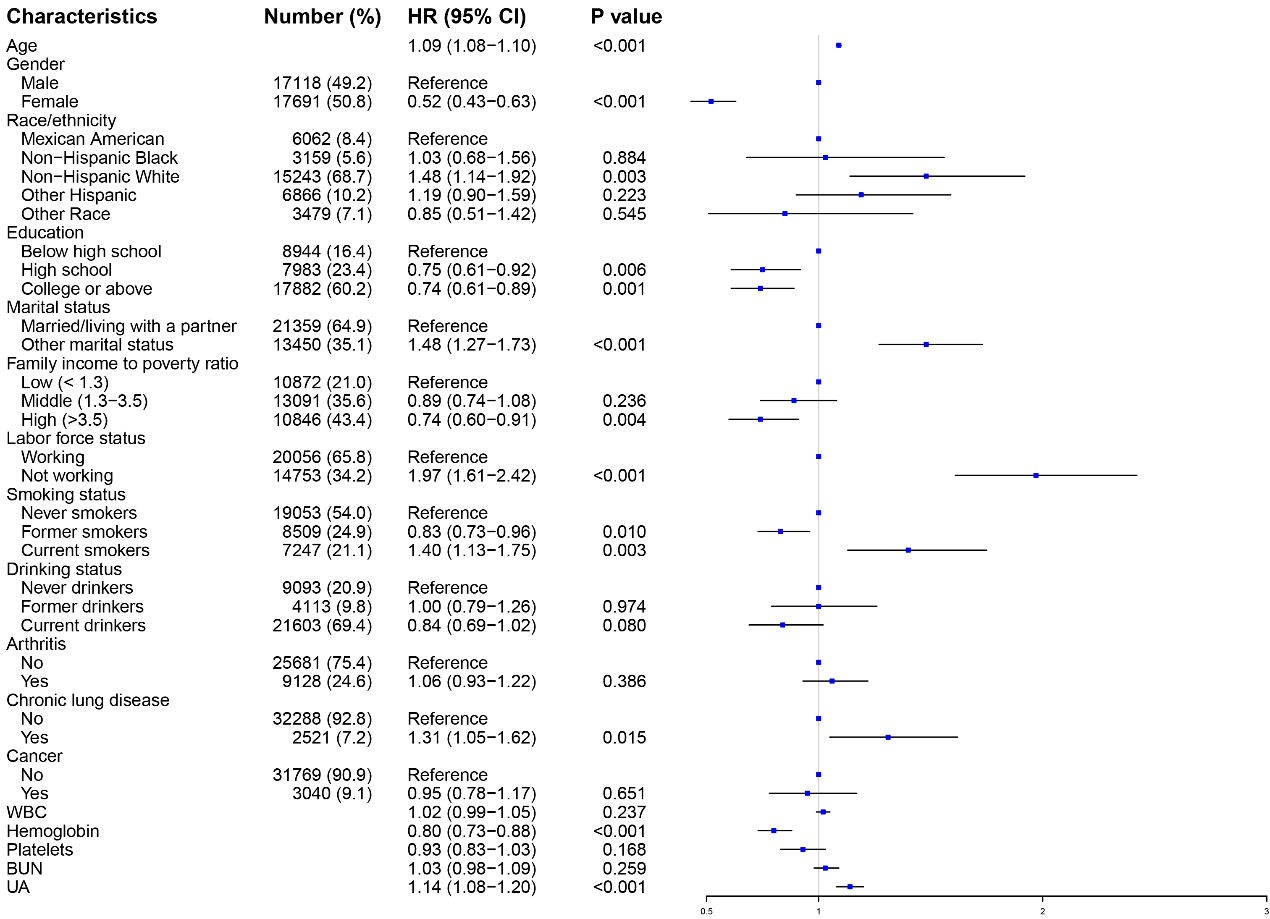


CKM, cardiovascular-kidney-metabolic; HR, hazard ratio; CI, confidence interval; WBC, white blood cell; BUN, blood urea nitrogen; UA, uric acid.
